# Detection of pre-existing SARS-CoV-2-reactive T cells in unexposed renal transplant patients

**DOI:** 10.1007/s40620-021-01092-0

**Published:** 2021-07-06

**Authors:** Moritz Anft, Arturo Blazquez-Navarro, Ulrik Stervbo, Sarah Skrzypczyk, Oliver Witzke, Rainer Wirth, Mira Choi, Christian Hugo, Petra Reinke, Toni Luise Meister, Eike Steinmann, Stephanie Pfaender, Peter Schenker, Richard Viebahn, Timm H. Westhoff, Nina Babel

**Affiliations:** 1 Center for Translational Medicine and Immune Diagnostics Laboratory, Medical Department I, Marien Hospital Herne, University Hospital of the Ruhr-University Bochum, Hölkeskampring 40, 44625 Herne, Germany; 2 Berlin Institute of Health at Charité – Universitätsmedizin Berlin, BIH Center for Regenerative Therapies (BCRT), Charitéplatz 1, 10117 Berlin, Germany; 3 Department of Infectious Diseases, West German Centre of Infectious Diseases, University Hospital Essen, University Duisburg-Essen, Hufelandstraße 55, 45147 Essen, Germany; 4 Department of Geriatrics, Marien Hospital Herne, University Hospital of the Ruhr-University Bochum, Hölkeskampring 40, 44625 Herne, Germany; 5 Department of Nephrology, Medical Department III, Universitätsklinikum Carl Gustav Carus, TU Dresden, Fetscherstraße 74, 01307 Dresden, Germany; 6 Department of Molecular and Medical Virology, Ruhr University Bochum, Universitätsstrasse 50, 44801 Bochum, Germany; 7 Department Surgery, Knappschaftskrankenhaus Bochum, University Hospital of the Ruhr-University Bochum, In der Schornau 23, 44892 Bochum, Germany

**Keywords:** Renal transplantation, Sars-CoV-2, Antigen-spcific T cells, Immunosuppression

## Abstract

**Background:**

Recent data demonstrate potentially protective pre-existing T cells reactive against the severe acute respiratory syndrome coronavirus 2 (SARS-CoV-2) in samples of healthy blood donors, collected before the SARS-CoV-2 pandemic. Whether pre-existing immunity is also detectable in immunosuppressed patients is currently not known.

**Methods:**

Fifty-seven patients were included in this case–control study. We compared the frequency of SARS-CoV-2-reactive T cells in the samples of 20 renal transplant (RTx) patients to 20 age/gender matched non-immunosuppressed/immune competent healthy individuals collected before the onset of the SARS-CoV-2 pandemic. Seventeen coronavirus disease 2019 (COVID-19) patients were used as positive controls. T cell reactivity against Spike-, Nucleocapsid-, and Membrane- SARS-CoV-2 proteins were analyzed by multi-parameter flow cytometry. Antibodies were analyzed by neutralization assay.

**Results:**

Pre-existing SARS-CoV-2-reactive T cells were detected in the majority of unexposed patients and healthy individuals. In RTx patients, 13/20 showed CD4^+^ T cells reactive against at least one SARS-CoV-2 protein. CD8^+^ T cells reactive against at least one SARS-CoV-2 protein were demonstrated in 12/20 of RTx patients. The frequency and Th1 cytokine expression pattern of pre-formed SARS-CoV-2 reactive T cells did not differ between RTx and non-immunosuppressed healthy individuals.

**Conclusions:**

This study shows that the magnitude and functionality of pre-existing SARS-CoV-2 reactive T cell in transplant patients is non-inferior compared to the immune competent cohort. Although several pro-inflammatory cytokines were produced by the detected T cells, further studies are required to prove their antiviral protection.

**Graphic abstract:**

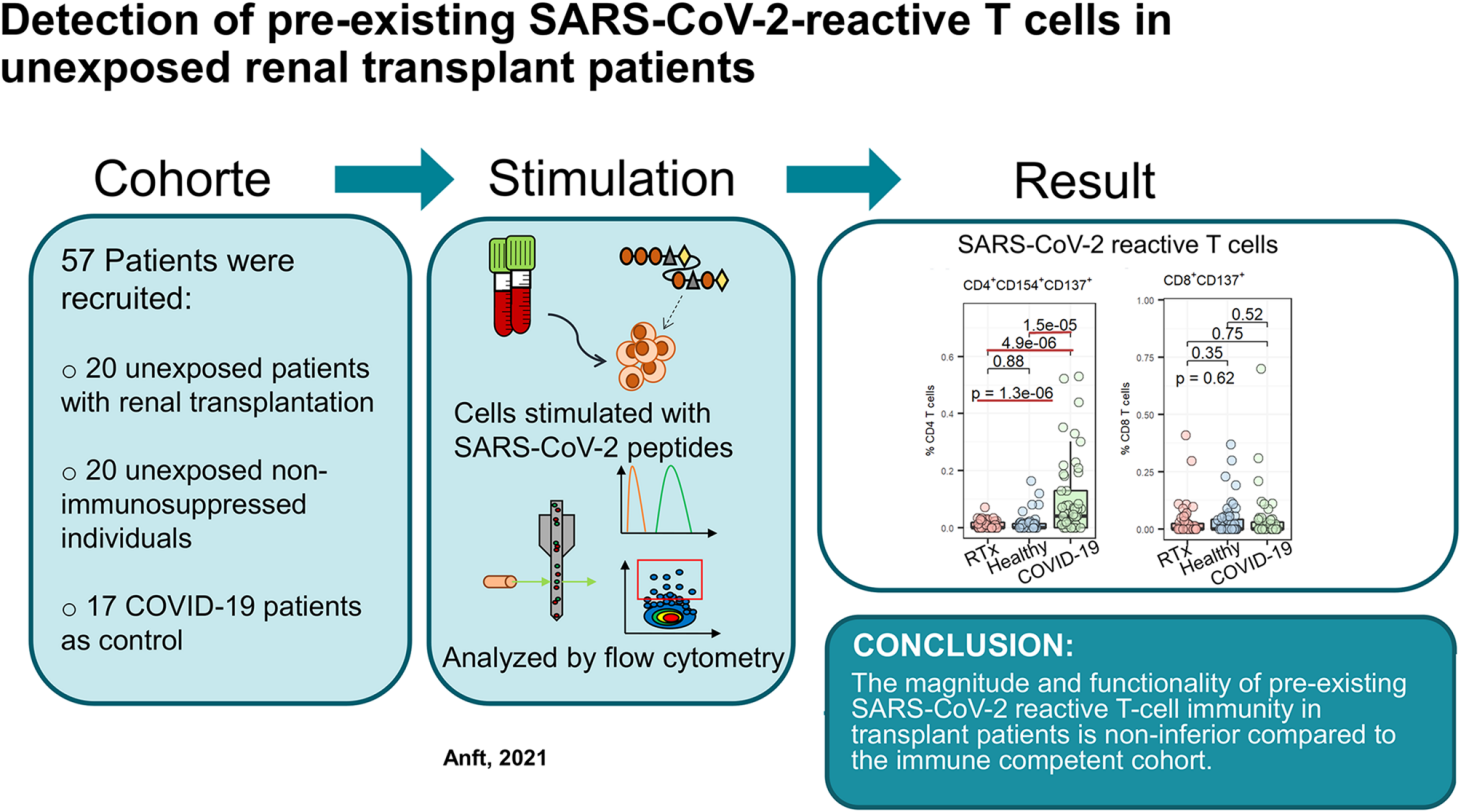

**Supplementary Information:**

The online version contains supplementary material available at 10.1007/s40620-021-01092-0.

## Introduction

The 2020 pandemic outbreak of the severe acute respiratory syndrome coronavirus 2 (SARS-CoV-2) resulted in over thirty million confirmed cases of coronavirus disease 2019 (COVID-19) and over two million associated deaths by January 2021 [1]. The clinical manifestation of COVID-19 is very heterogeneous, ranging from asymptomatic to cases with acute respiratory distress syndrome (ARDS) followed by multi-organ failure [2]. These severe courses mostly emerge with high levels of pro-inflammatory cytokines in a so-called cytokine storm [3] and the outcome deteriorates with increasing age and the existence of underlying health conditions [4].The immune response against SARS-CoV-2 leads to the generation of T cells, which specifically recognize peptides of the structural proteins: spike glycoprotein (S), the envelope (E) protein, the membrane (M) protein and the nucleocapsid (N) protein [5–7]. An insufficient immune response, due to weak antigen presentation or insufficient functionality of SARS-CoV-2-reactive T cells, has been suggested to cause critical COVID-19 [6, 8, 9]. On the other hand, an excessive immune response has also been associated with a critical COVID-19 disease course [10].

Seven of the known members of the coronavirus family can infect humans: the four common cold viruses (229-E, NL63, OC43, HKU1) and the less common Middle East Respiratory Syndrome (MERS)-CoV, SARS-CoV and SARS-CoV-2 (reviewed in [11]). Studies investigating the immunizing effect of infections with human coronaviruses are rare but there is first evidence supporting a long term immunizing effect after SARS infection [12, 13]. In contrast, the immunizing effect is shorter after infection with endemic coronaviruses, which makes reinfection with the same endemic coronavirus possible within a year [14]. While investigating blood samples collected before the pandemic, it became clear that healthy individuals with no prior contact with the virus harbor SARS-CoV-2 reactive T cells [6, 7, 15, 16]. These T cells probably arise due to infections with endemic coronaviruses which have recently been associated with less severe COVID-19 [17]. Because of the sequence similarities, endemic coronaviruses are the most likely cause of this cross-reactivity [18]. Although not demonstrated for SARS-CoV-2, cross-reactive T cells have also been shown to be proper functional and are able to protect against SARS and MERS infection in mouse models [19].

Immunosuppression is essential to prevent graft rejection in patients after transplantation [20]. However, immunosuppression can also inhibit the establishment of efficacious antiviral immunity that is crucial for effective antiviral protection and virus clearance [21–23]. While the presence of pre-existing SARS-CoV-2 T cell immunity has been demonstrated in several studies on the general population, the functionality of these presumably protective SARS-CoV-2 cross-reactive T cells in immunosuppressed patients remains unclear.

Furthermore, studies on the outcome of COVID-19 in transplant patients are contradictory: Pereira et al. reported that transplanted patients exhibit a higher rate of severe courses of COVID-19 [24], and a study in New York showed drastically increased morbidity of 28% compared to 1–5% in the general population [25]. In contrast, a study by Becchetti et al. showed no differences in the outcome after COVID-19 in transplanted and non-transplanted patients [26]. Thus, it remains unresolved whether transplant patients are at greater risk of suffering a severe or critical COVID-19 course.

The aim of the current study is to elucidate the quantity and quality of pre-existing SARS-CoV-2 reactive T cells in immunosuppressed renal transplant (RTx) patients and compare the found immunity to the matched cohort of non-immunosuppressed patients. To this end, we utilized biobanked peripheral blood mononuclear cells (PBMCs) from patients recruited before the pandemic to guarantee no prior infection with SARS-CoV-2.

## Methods

### Study population and design

Forty SARS-CoV-2 unexposed immunosuppressed (n = 20) and non-immunosuppressed (n = 20) healthy individuals were recruited into the study prior to the SARS-CoV-2 pandemic. As a positive control, 17 hospitalized patients in the resolution phase of convalescent SARS-CoV-2 infection were analyzed shortly before discharge. No COVID-19 patients with critical disease were included. The cohorts showed no differences in sex (Supplementary Table 2), but COVID-19 patients were significantly older compared to RTx patients and healthy individuals (Supplementary Fig. 2), and 11/17 COVID-19 patients had lymphopenia. Renal transplant patients were treated with immunosuppression as depicted in Supplementary Table 3, and underwent transplantation 2–9 months prior to analysis. None of the RTx patients received T cell depletion therapy.

### Study approval

The study was approved by the ethical committee of the Ruhr-University Bochum (20-6886) and University Hospital Essen (20-9214-BO). Written informed consent was obtained from all participants.

### Antibodies

All antibodies are from BioLegend, USA unless otherwise noted: surface staining: CCR7 (CD197)-PerCP-Cy5.5; clone: G043H7, CD4-A700; clone: OKT4, LD eFluor780 (eBioscience, USA), CD8-V500; clone: RPA-T8 (BD Biosciences, USA), CD45RA-BV605; clone: HI100. Intracellular staining: granzyme B-FITC; clone: GB11, IL-2-PE; clone: MQ1-17H12, CD137 (4-1BB)-PE-Cy7; clone: 4B4-1, CD154 (CD40L)-A647; clone: 24–31, TNFα-eFluor450; clone: MAb11 (eBioscience, USA), IFNg-BV650; clone: 4S.B3, CD3-BV785; clone: OKT3. Fixable Viability Dye eFluor 780(eBioscience, USA) was used for live/dead discrimination.

### Preparation of PBMCs and stimulation with SARS-CoV-2 overlapping peptide pools

Peripheral blood mononuclear cells (PBMCs) were prepared from whole blood by gradient centrifugation as previously described [27]. Isolated PBMCs were stimulated with 15mer overlapping peptide pools (OPPs) from SARS-CoV-2 proteins with an overlap of 11 amino acids. SARS-CoV-2 PepTivator peptide pools (Miltenyi Biotec, Germany) were used containing overlapping peptides spanning in silico predicted immune dominant parts of the S-protein [28], or, covering the complete sequence of the N- and M-proteins. Peptide pools were dissolved per the manufacturer’s instructions in sterile water and used at a concentration of 1 µg/ml. 2.5 × 10^6^ PBMCs were plated for each condition in 96-U Well Plates in RPMI media (Life Technologies, USA), supplemented with 1% Penicillin–Streptomycin–Glutamine (Sigma Aldrich, USA) and 10% FCS (PAN-Biotech, Germany) and were stimulated or left untreated as a control for 16 h. As a positive control, cells were stimulated with SEB (1 µg/ml, Sigma Aldrich) and negative control was with vehicle (a medium to dissolve peptide pools). After 2 h Brefeldin A (5 µg/ml, Sigma Aldrich, USA) was added. T cells stimulated with SARS-CoV-2 OPPs were stained with optimal concentrations of antibodies for 10 min at room temperature in the dark. Stained cells were washed twice with PBS/BSA before preparation for intracellular staining using the Intracellular Fixation & Permeabilization Buffer Set (Thermo Fisher Scientific, USA) as per the manufacturer’s instructions. Fixed and permeable cells were stained for 30 min at room temperature in the dark with optimal dilution of antibodies against intracellular antigens. All samples were immediately acquired on a CytoFlex flow cytometer (Beckman Coulter, USA). Antigen-specific responses were considered positive after non-specific background was subtracted and more than 0.01% cells were positive. Negative values were set to zero. Quality control was performed daily using the recommended CytoFLEX Daily QC Fluorospheres (Beckman Coulter, USA). No modification to the compensation matrices was required throughout the study. As a result of low cell counts after thawing, only PBMCs from 11/20 renal transplantation patients and 19/20 healthy controls could be stimulated with the M protein.

### Neutralizing antibody

For the virus neutralization assay, sera were incubated for 30 min at 56 °C in order to inactivate complement factors. A propagation-incompetent VSV*ΔG(FLuc) pseudovirus system bearing the SARS-CoV-2 spike protein in the envelope was incubated with quadruplicates of twofold serial dilutions of immune sera in 96-well plates prior to infections of Vero E6 cells (1 × 10^4^ cells/well) in DMEM + 10% FBS (Life Technologies, USA). At 18 h post infection, firefly luciferase (FLuc) reporter activity was determined after adding 25 µL of firefly luciferase ONE-GloTM substrate (Promega, USA) using a GloMax plate reader (Promega, USA) and the reciprocal antibody dilution causing 50% inhibition of the luciferase reporter calculated (PVND50).

### Statistical analysis

Flow cytometry data were analyzed using FlowJo version 10.6.2 (BD Biosciences, USA); gating strategies are presented in Figs. 2a and 5a. Statistical analysis was performed using R, version 3.6.2. Box plots depict the median, first and third quartile of a variable; the maximum length of the whiskers corresponds to 1.5 times the interquartile range. The applied statistical tests are two-sided. Differences between three groups for categorical variables were calculated using the Kruskal–Wallis test, and pairwise comparison between two groups were analyzed using the post-hoc unpaired, two-tailed Mann–Whitney *U* test. p-values below 0.050 were considered significant and are underlined in red. Descriptive statistics are indicated as median [min—max].

## Results

### Immunosuppressed renal transplant patients have detectable SARS-CoV-2 reactive T cells without prior virus exposure

The existence of cross reactive SARS-CoV-2 T cells in individuals without prior contact with the virus could result in protective background immunity in the human population. It is of interest if immunosuppressed transplant patients have detectable SARS-CoV-2 reactive T cells and therefore might also benefit from pre-existing immunity. To address this question, we analyzed 20 samples obtained from RTx patients and compared them with a matched cohort of 20 non-immunosuppressed healthy individuals. All samples had been frozen before the onset of the SARS-CoV-2 pandemic, which ensures no prior exposure to SARS-CoV-2. The isolated PBMCs were treated with an overlapping peptide pool (OPP) containing peptides spanning the N and M proteins or the immunodominant parts of the S protein. Using multi-parameter flow cytometry analysis, the frequencies of SARS-CoV-2 reactive T cells were assessed according to the expression of the activation markers after the background subtraction from unstimulated cell samples. Thus, antigen-specific T helper cells were defined as CD4^+^CD154^+^CD137^+^, and antigen-specific cytotoxic T cells as CD8^+^CD137^+^ (Fig. 2a, [29]). Negative values or values below a threshold of 0.01% were set to zero.

**Fig. 1:**
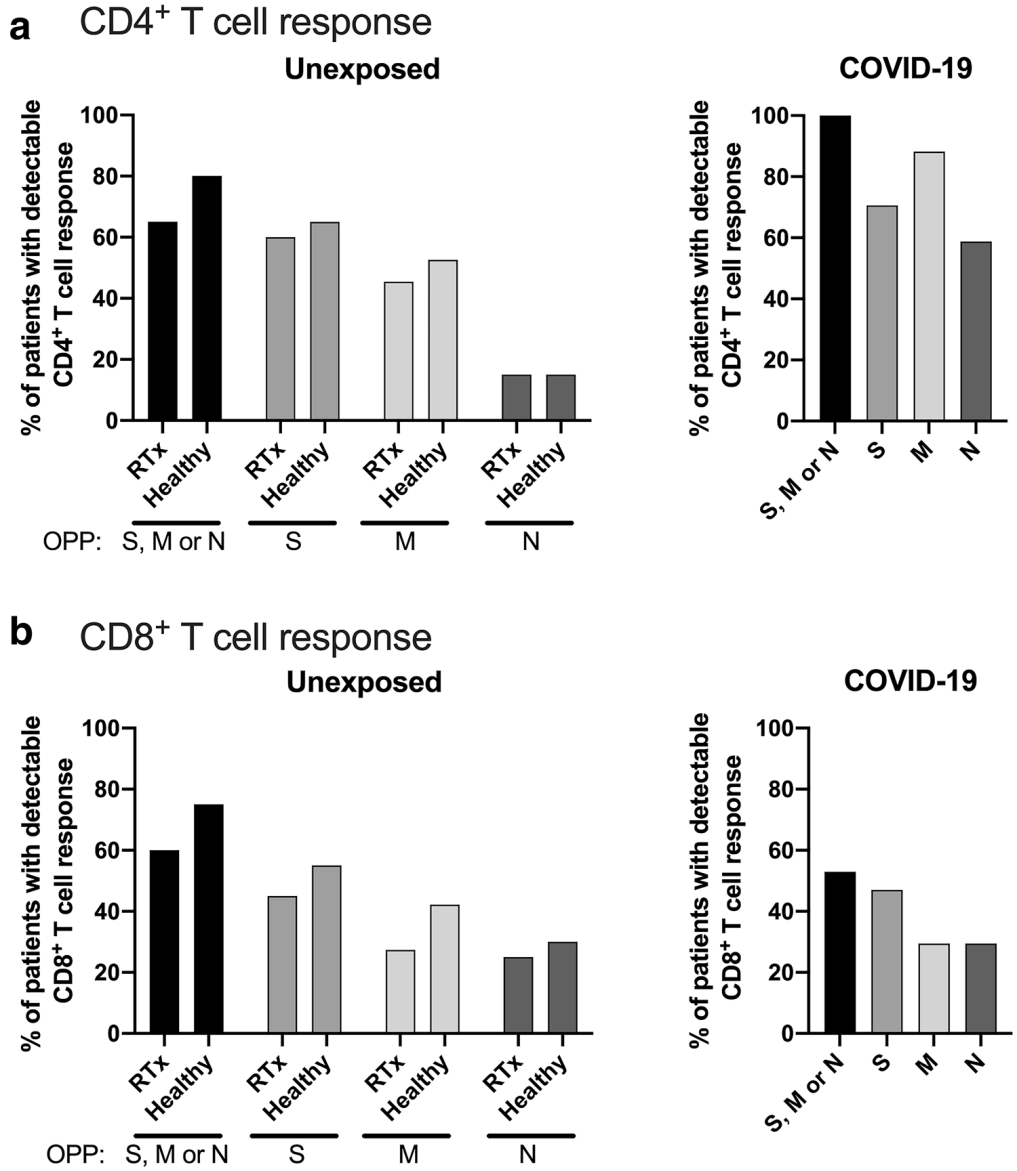
T cell response in unexposed and COVID-19 patients. Percentage of unexposed RTx patients, healthy or exposed COVID-19 patients with a detectable CD4 (**a**) or CD8 (**b**) T cell response after stimulation with S, M or N OPPs. A detectable CD4 and CD8T cell response was defined as a minimum of 0.01% CD4^+^CD154^+^CD137^+^ or CD8^+^CD137^+^ T cells after background of unstimulated cells was subtracted

**Fig. 2:**
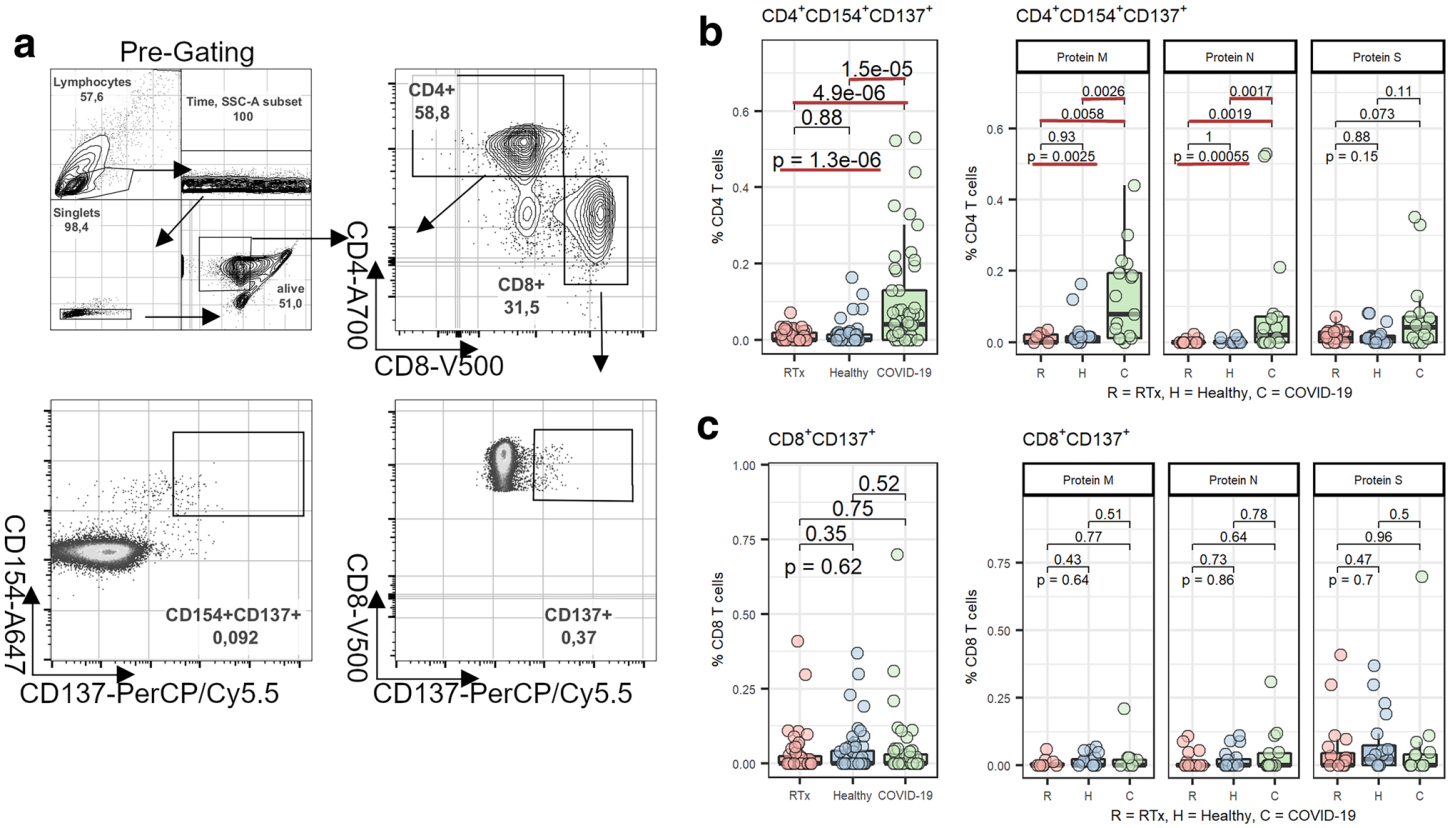
Pre-existing SARS-CoV-2 reactive T cells can be detected in renal transplant patients. Isolated PBMCs from RTx patients (20), healthy individuals (20) and COVID-19 patients (17) were stimulated for 16 h with 1 µg/ml SARS-CoV-2 OPPs from M (n = 11–19), N (n = 17–20) or S (n = 17–20) proteins and afterwards stained with antibodies against the depicted markers for flow cytometry analysis. **a** Antigen-specific T helper cells were identified as Live/Dead-Marker^−^CD3^+^CD4^+^CD137^+^CD154^+^ and antigen-specific cytotoxic T cells were identified as Live/Dead-Marker^−^CD3^+^CD8^+^CD137^+^. Frequency of overall SARS-CoV-2 reactive CD4^+^CD137^+^CD154^+^ (**b**, left panel) and CD8^+^CD137^+^ (**c**, left panel) T cells specific to the M, N or S protein combined and subdivision in cells reactive towards the M, N or S protein among all CD4^+^ or CD8^+^ cells, respectively. Groups were compared using the Kruskal–Wallis test (indicated by “p- = ”); pairwise comparison was done using the two-sided, unpaired post-hoc Mann–Whitney *U* test. p-values < 0.05 are underlined in red

We were able to detect SARS-CoV-2 reactive CD4^+^ T cells in both RTx patients and healthy individuals with no prior contact to the virus after stimulation with tbe M protein (45% and 53%, respectively), N protein (15% and 15%, respectively) or S protein (60% and 65%, respectively)(Fig. 1a). Additionally, we also found detectable SARS-CoV-2 reactive CD8^+^ T cells in RTx patients and in healthy individuals after stimulation with the M protein (27% and 42%, respectively), N protein (25% and 30%, respectively) or S protein (45% and 55%, respectively) (Fig. 1b). Notably, we found no differences in any cohort characteristics between RTx patients with or without a CD4^+^ or CD8^+^ T cell response (Supplementary Table 1). While investigating the magnitude of the combined CD4^+^ T cell responses against the SARS-CoV-2 M, N and S proteins, we found a similar immune response in RTx patients and healthy individuals (Fig. 2b, left panel). Moreover, the responses towards the individual proteins were also comparable and did not differ significantly between RTx patients and healthy individuals (Fig. 2b, right panel). To compare the immune response of the pre-formed cross reactive T cells of unexposed individuals to T cells derived from patients during COVID-19 resolution, we also analyzed 17 COVID-19 patients shortly before discharge. As expected, the CD4^+^ T cell responses from patients with COVID-19 were significantly stronger for all three proteins combined and for the individual M and N proteins, but interestingly, not for the S protein (Fig. 2b). Additionally, the magnitude of the CD8^+^ T cell immune responses was similar for RTx patients and healthy individuals after stimulating with all tested SARS-CoV-2 proteins. But in contrast to CD4^+^ T cells, the CD8^+^ immune responses of both unexposed cohorts did not significantly differ from patients with COVID-19 (Fig. 2c).

Taken together, immunosuppressed RTx patients are able to generate SARS-CoV-2 cross reactive T cells, which are comparable to those of healthy blood donors but with a clearly lower frequency of CD4^+^ T cells reactive to M and N proteins compared to COVID-19 patients.

### Polyfunctional SARS-CoV-2 cross-reactive T cells can be detected in RTx patients and healthy individuals with a comparable response magnitude

**Fig. 3:**
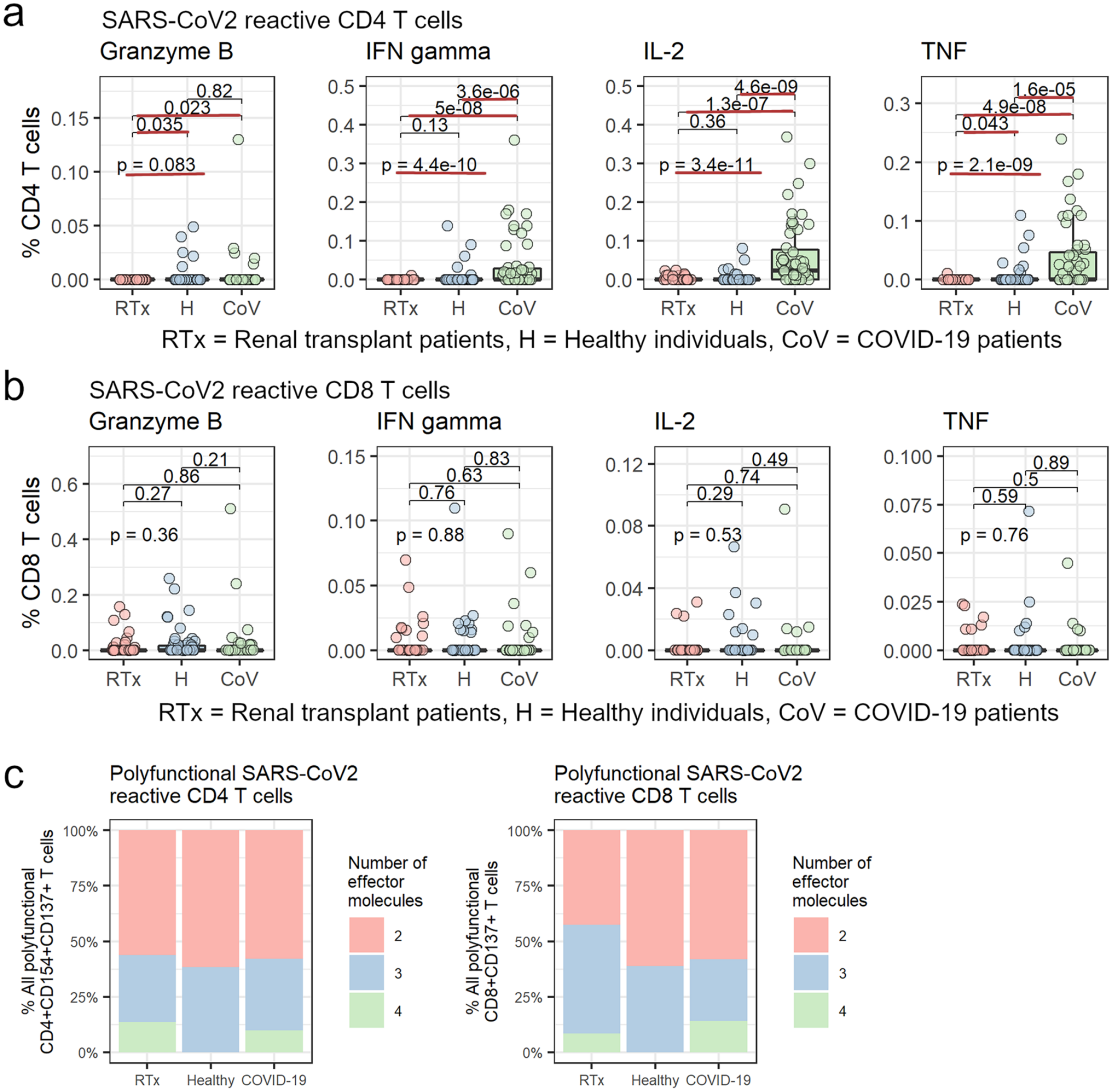
RTx patients and healthy individuals have equal frequencies of pre-existing SARS-CoV-2 reactive T cells expressing Th1 cytokines or granzyme B. Isolated PBMCs from RTx patients (20), healthy individuals (20) and COVID-19 patients (17) were stimulated for 16 h with 1 µg/ml SARS-CoV-2 OPPs from M (n = 11–19), N (n = 17–20) or S (n = 17–20) proteins. Expression of Th1 cytokines IFNγ, IL-2 or TNF and granzyme B in antigen-specific CD4^+^CD137^+^CD154^+^ **a** and CD8^+^CD137^+^ **b** among all CD4 + or CD8 + cells, respectively. **c** Fraction of patients with SARS-CoV-2 reactive T cells co-expressing 2–4 cytokines or granzyme b among all antigen-specific T cells. Groups were compared using the Kruskal–Wallis test (indicated by “p- = ”); pairwise comparison was done using the two-sided, unpaired post-hoc Mann–Whitney *U* test. p-values < 0.05 are underlined in red. (*H* healthy individuals, *CoV* COVID-19 patients)

Besides its presence in the circulation, the functional activity of SARS-CoV-2-specific T cells is crucial for antiviral control. Two recent papers demonstrated a correlation between the frequencies of IFNγ-producing SARS-CoV-2-reactive T cells and COVID-19 severity [9, 29]. Therefore, we analyzed the frequency of SARS-CoV-2 T cells expressing pro-inflammatory Th1 cytokines IFNγ, IL-2, and TNF together with the cytotoxic granzyme B after stimulation with SARS-CoV-2 OPPs. Using frequencies of IFNγ-producing T cells as a potential measure of antiviral protection, we compared the SARS-CoV-2-reactive T cell response between the analyzed groups. We detected IFNγ-expressing SARS-CoV-2-reactive CD4^+^ T cells in only 5% of RTx patients and in 20% of healthy individuals, but in 59% of COVID-19 patients. The magnitude of IFNγ and IL-2 producing SARS-CoV-2 CD4^+^ T cell response was also not significantly different between the two groups of unexposed patients. Interestingly, the magnitude of TNF and granzyme B producing SARS-CoV-2 reactive CD4^+^ T cells was significantly higher in healthy individuals compared to RTx patients. As expected, the magnitude of IFNγ, IL-2 and TNF producing effector cells was significantly elevated in COVID-19 patients (Fig. 3a). In contrast to CD4^+^ T cells, CD8^+^ T cell response to SARS-CoV-2 OPPs showed a different pattern. Although some RTx patients and healthy individuals showed strong cytokine production by CD8^+^ T cells after stimulation with OPPs, the magnitude of CD8^+^ T cell response was in general lower for all measured cytokines and did not differ significantly between RTx patients and healthy individuals. Remarkably, the cytokine producing CD8^+^ T cell immune response of RTx patients and healthy individuals was also not significantly different compared to what was observed in COVID-19 patients (Fig. 3b). To further analyze the functional capacity of the SARS-CoV-2 reactive T cells, we quantified the frequency of polyfunctional T cells, which are known to bear the highest antiviral protection [30]. We were able to detect CD4^+^ and CD8^+^ polyfunctional antigen-specific T cells, expressing two, three or four Th1 cytokines or granzyme B in RTx patients and healthy individuals (Fig. 3c). The frequency of bi-, tri- and tetrafunctional T cells among all antigen-specific T cells was similar for RTx patients and healthy individuals and did not differ compared to COVID-19 patients either (Fig. 3c).

Although measurable functional active T cell immunity was detected in RTx and healthy controls, SARS-CoV-2 neutralizing antibodies were not detectable in unexposed patients. Taken together, a functionally active SARS-CoV-2 cross-reactive T cell response can be generated in transplant patients, despite immunosuppression. This Th1 cytokine or granzyme B producing T cell response is comparable to what is seen healthy individuals but clearly inferior to COVID-19 patients.

### Pre-formed SARS-CoV-2 reactive T cells demonstrate an immunodominant response against the S and M proteins

**Fig. 4:**
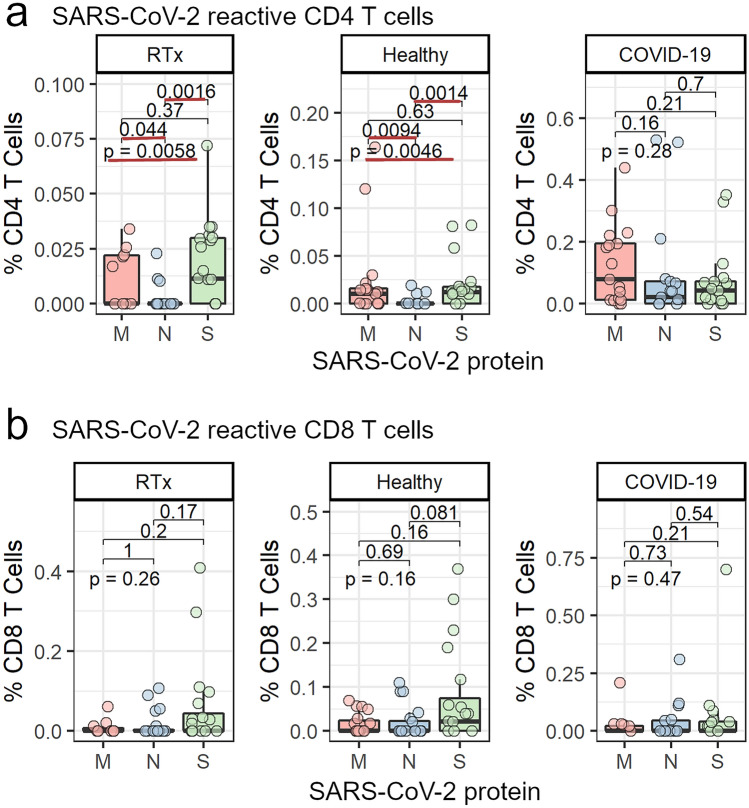
Differences in the frequency of SARS-CoV-2 M, N or S protein reactive T cells. Isolated PBMCs from RTx (20), healthy individuals (20) and COVID-19 patients (17) were stimulated for 16 h with 1 µg/ml SARS-CoV-2 OPPs from M (n = 11–19), N (n = 17–20) or S (n = 17–20) proteins. Comparison of the frequencies of SARS-CoV-2 reactive **a** CD4^+^CD137^+^CD154^+^ and **b** CD8^+^CD137^+^ T cells after stimulation with the M, N or S proteins in RTx patients and healthy individuals. Groups were compared using the Kruskal–Wallis test (indicated by “p- = ”); pairwise comparison was done using the two-sided, unpaired post-hoc Mann–Whitney *U* test. p-values < 0.05 are underlined in red

After the 2003 SARS pandemic, studies demonstrated that SARS-CoV-reactive CD4^+^ T cells show a dominant immune response against the S protein compared with N or M proteins [31]. In the next step, we wanted to investigate if there is an immune dominant restriction towards one of the three tested SARS-CoV-2 proteins for unexposed individuals. The magnitude of the pre-formed SARS-CoV-2 reactive CD4^+^ cells in RTx and healthy individuals was significantly increased after challenging with the M and S proteins compared to the N protein. In contrast, COVID-19 patients showed no immunodominant restriction towards any of the three tested SARS-CoV-2 proteins (Fig. 4a). In contrast, we did not find a dominant reaction towards any of the tested proteins in patients with detectable pre-formed antigen-specific CD8^+^ T cells or in COVID-19 patients (Fig. 4b). These results demonstrate that RTx patients and healthy individuals show similar immunodominant restriction within three tested SARS-CoV-2 proteins with a superior immune reaction towards the S and M proteins in contrast to COVID-19 patients, as reflected by the frequency of activation marker positive T cells.

### Similar phenotypic differential stage of SARS-CoV-2-reactive T cells in RTx patients and healthy controls

**Fig. 5:**
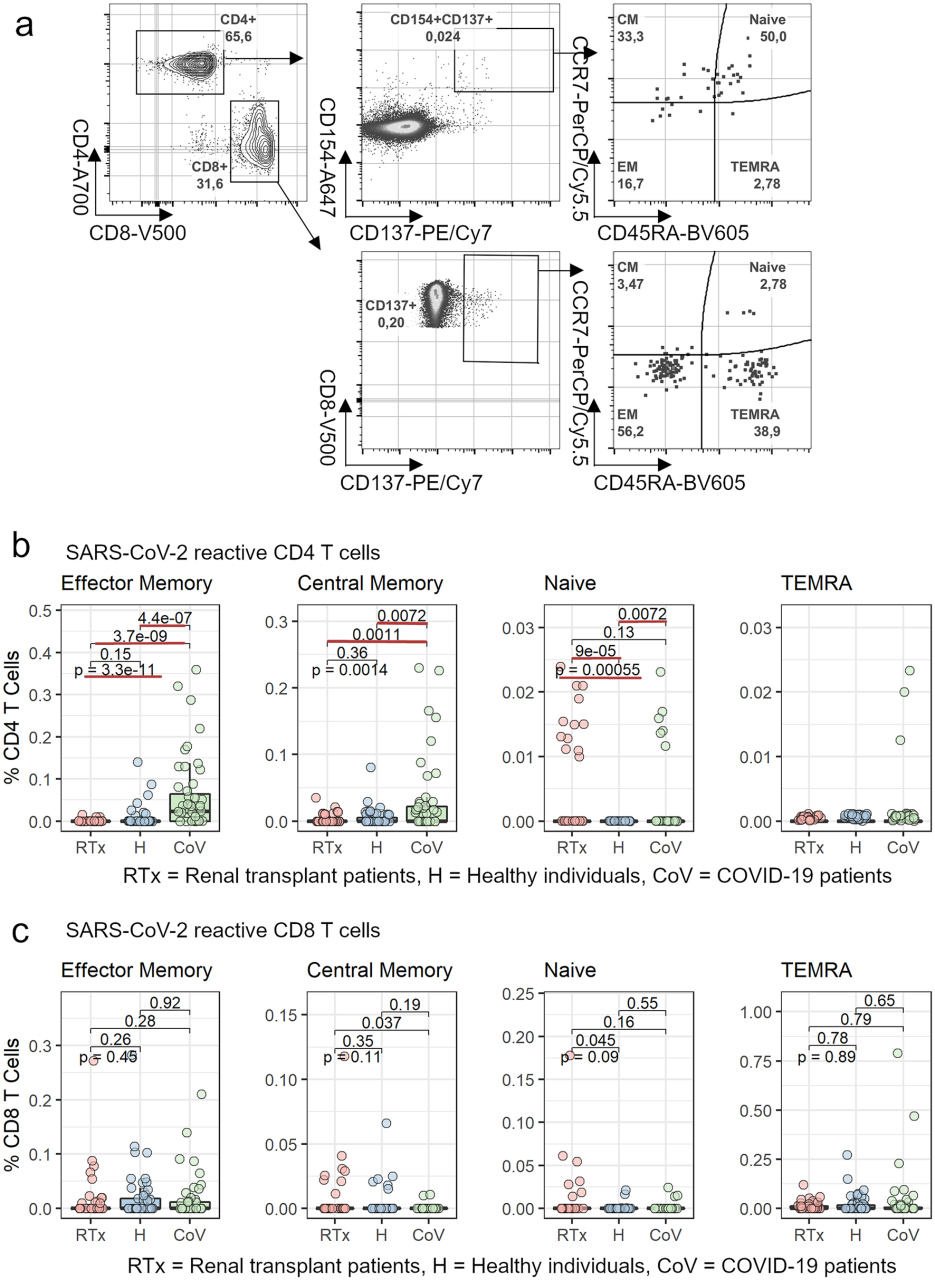
Comparison of SARS-CoV-2 reactive memory cell phenotypes. Isolated PBMCs from RTx patients (20), healthy individuals (20) and COVID-19 patients (17) were stimulated for 16 h with 1 µg/ml SARS-CoV-2 OPPs from M (n = 11–19), N (n = 17–20) or S (n = 17–20) proteins and analyzed by flow cytometry. **a** Identification of antigen-specific memory T cells: After gating on SARS-CoV-2 reactive CD4^+^CD137^+^CD154^+^ and CD8^+^CD137^+^ T cells, memory cells were identified by expression of CD45RA and CCR7 as naïve (CD45RA^+^CCR7^+^), central memory (CM, CD45RA^−^CCR7^+^), effector memory (EM, CD45RA^−^CCR7^−^) and TEMRA (CD45RA^+^CCR7^−^) cells. Comparison of SARS-CoV-2 reactive memory **b** CD4^+^CD137^+^CD154^+^ and **c** CD8^+^CD137^+^ T cells in RTx patients, healthy individuals and COVID-19 patients. Groups were compared using the Kruskal–Wallis test (indicated by “p- = ”); pairwise comparison was done using the two-sided, unpaired post-hoc Mann–Whitney *U* test. p-values < 0.05 are underlined in red.(*H* healthy individuals, *CoV* COVID-19 patients)

In mouse models, cross-reactive CD4^+^ central memory cells, generated after a SARS-CoV infection, have been shown to induce a protective T cell immune response against MERS infection [6]. In the next step, we wanted to investigate whether unexposed RTx patients and healthy individuals harbor potentially protective SARS-CoV-2 memory T cells. Using CD45RA and CCR7 markers which allow to distinguish between antigen reactive CD4^+^ and CD8^+^ naïve, effector memory (EM), central memory (CM) and T effector memory RA (TEMRA) cells (Fig. 5a), we assessed the differential status of SARS-CoV-2-reactive T cells. By analyzing SARS-CoV-2 reactive CD4^+^ T memory cell subsets, we found that RTx patients and healthy individuals did not differ with regard to the frequency of effector memory, central memory or TEMRA cells. Notably, the frequency of naïve CD4^+^ T cells, reactive to SARS-CoV-2, was significantly increased in RTx patients compared to healthy individuals (Fig. 5b). In contrast, patients with COVID-19 had significantly more effector and central memory CD4^+^ T cells compared to RTx patients and healthy individuals and an equal frequency of naïve CD4^+^ T cells compared to RTx patients (Fig. 5b). The CD8^+^ T cell memory response was similar for RTx patients and healthy donors and did not differ in any memory or naïve phenotype (Fig. 5c). Comparing the frequencies of antigen-specific memory T cells reactive to the individual M, N or S proteins, we also found significantly fewer CD4^+^central memory T cells specific for the N protein compared to the M and S proteins in RTx patients and healthy individuals, but no differences for CD8^+^ memory T cell subsets (Supplementary Fig. 1).

These results show that immunosuppressed renal transplant patients are able to develop SARS-CoV-2 reactive memory T cells with a magnitude and differentiation phenotype comparable to healthy individuals. Nevertheless, COVID-19 patients show clearly elevated frequencies of CD4^+^ memory T cells.

## Discussion

The 2020 SARS-CoV-2 pandemic has provoked enormous challenges for health care systems and economies all over the world. The lack of antiviral therapies makes it indispensable to identify risk groups in order to protect them from infections, which otherwise could result in a severe COVID-19 course. One potential high-risk group are transplant patients, as they need immunosuppressive treatment and often suffer co-morbidities like hypertension, cardiovascular diseases, diabetes or malignancies, which worsen the COVID-19 course [32]. An advantage in the control of COVID-19 could be protective background immunity caused by cross-reactive T cells, which is already known for SARS-CoV, the coronavirus strain responsible for the 2003 SARS pandemic [33]. In this study, we addressed the question of whether immunosuppressed renal transplant patients are able to generate and mount cross-reactive immunity against SARS-CoV-2. Immunosuppression in patients suffering from COVID-19 is a double-edged sword. Given the importance of a highly functional immune system in order to prevent and fight infections, immunosuppression can extenuate the immune response. This can lead to a severe course of disease as shown for CMV [34] or BKV [23] infections. On the other hand, an acute SARS-CoV-2 infection with critical course is linked to immunopathogenesis [10], and overwhelming immune reactions can be moderated by immunosuppressive drugs, as has been discussed for the IL-6 neutralizing antibody, tocilizumab [35] or the corticoid, dexamethasone [36]. First case reports on heart [37] or renal [38] transplant patients show the possibility of controlling an acute COVID-19 infection, even when the patients remain on tacrolimus or mycophenolate mofetil (MMF). Nevertheless, in some cases, severe complications have been reported for transplant patients with SARS-CoV-2 infection [39, 40]. The influence of immunosuppressive drugs on the development of pre-existing and cross-reactive immune cells prior to a SARS-CoV-2 infection has not yet been investigated.

Herein we show that renal transplant patients are able to develop SARS-CoV-2 cross-reactive T cells despite undergoing treatment with immunosuppressants such as tacrolimus, glucocorticoids and MMF. Moreover, the frequency of the detected cross-reactive T cells was comparable to the number detected in unexposed healthy individuals. This shows that immunosuppression does not inhibit the development of SARS-CoV-2 cross-reactive immunity. The frequency of SARS-CoV-2 cross-reactive T cells in unexposed individuals was found to differ in recent studies from 20 to 80% [7, 16, 18, 41, 42] depending on origin, age and methodological differences in the study design. In our study we found that 60–80% of the unexposed RTx patients or healthy individuals have cross-reactive SARS-CoV-2 CD4^+^ or CD8^+^ T cells reactive against at least one of the three tested proteins, i.e., M-, N- or S. But different T cell stimulation assays, overlapping peptide pools and cut-offs make it difficult to compare these numbers with other studies.

It is crucial that cross-reactive T cells are not only detectable but also functional in order to protect against SARS-CoV-2. In a recent study we showed that COVID-19 patients generate a large number of polyfunctional antigen-specific T cells, expressing at least two of the pro-inflammatory Th1 cytokines TNF, IL-2, IFNγ or the cytotoxic granzyme B, after stimulation with M, N or S OPPs [5]. Although cross-reactive CD4^+^ T cells found in RTx patients showed a lower magnitude compared to patients after COVID-19 infection, the singular or even simultaneous expression of IFNγ and other effector molecules indicates the potentially protective capacity of the CD4^+^ T cells against SARS-CoV-2. Importantly, RTx patients and healthy individuals showed no differences in cytokine-producing cross-reactive T cells, suggesting that the functional immune response is not altered by immunosuppression.

Another characterization of the protective potential of the detected cross-reactive T cells is their memory differentiation phenotype. In a mouse model, airway-derived CD4^+^ central memory T cells were shown to mediate protection against SARS-CoV in an IFNγ-dependent manner [19]. We found RTx patients and healthy individuals bearing SARS-CoV-2 cross-reactive memory CD4^+^ T cells, thus making it possible that these T cells can also mediate protection against the novel coronavirus. These findings are strengthened by the observation that the detected SARS-CoV-2 specific CD8^+^ T cells have a central memory, effector memory or TEMRA phenotype, which can exhibit T cell effector functions in RTx patients [43]. However, compared to COVID-19 patients, in RTx patients the CD4^+^ memory T cells were decreased and failed to produce cytokines to the same extent after challenging with SARS-CoV-2 OPPs. The protective capacity of these cells in a clinical setting needs further investigation.

It is striking that unexposed RTx patients and healthy individuals have a similar frequency of SARS-CoV-2-specific CD8^+^ T cells compared to COVID-19 patients. Although we analyzed CD8^+^ T cell response in mild and moderate COVID-19 patients, which are lower compared to patients with a critical course of infection [10], we would expect a clearly increased frequency in acutely infected patients compared to unexposed individuals. The identical frequencies of memory and naïve CD8^+^ T cells in COVID-19 patients compared to unexposed RTx patients and healthy individuals may be caused by the migration of SARS-CoV-2 CD8^+^ T cells into the infected tissues in COVID-19 patients with the subsequent depletion of these cells from the circulation.

Recent studies showed that cross-reactivity is not restricted to T cells but that it is also observed in humoral immune response, and that cross-reactive antibodies between coronaviruses are common [44]. Although in some cases these antibodies have a neutralizing capacity [45], in most cases they are not able to neutralize other viruses [46]. In line with these results, we did not detect any SARS-CoV-2 neutralizing antibodies in the serum of unexposed renal transplant patients or in healthy individuals.

Noteworthy, this study has some limitations. The investigated cohort in this study underwent transplantation 2–9 months prior to analysis, which also makes it theoretically possibile that the cross-reactive T cells were generated before the transplantation and start of immunosuppressive therapy.

In conclusion, this study demonstrates that RTx patients with no previous SARS-CoV-2 exposure are able to develop SARS-CoV-2 cross-reactive T cells with the magnitude and functionality comparable to unexposed non-immunosuppressed individuals. We were able to show that immunosuppression after transplantation has no negative effect on the generation of SARS-CoV-2 cross-reactive T cells. Importantly, the cross-reactive T cells of both unexposed cohorts were inferior in number and function as compared to patients after COVID-19 infection. Therefore, further studies are required to demonstrate the protective capacity of the detected pre-existing T cells in transplant populations.

## Supplementary Information

Below is the link to the electronic supplementary material.

Supplementary file1 (PDF 915 KB)

## Data Availability

Data is available on request from the authors.

## Code availability

No new code was produced in this study.
