# Supplementary material for: Detection of pre-existing SARS-CoV-2-reactive T cells in unexposed renal transplant patients
Source: J Nephrol. 2021 Jul 6;34(4):1025–37. doi: 10.1007/s40620-021-01092-0 (PMC8259083; doi:10.1007/s40620-021-01092-0)
Supplement: Supplementary file 1 — Supplementary file1 (PDF 915 KB) [file 40620_2021_1092_MOESM1_ESM.pdf]

Supplementary Appendix

Contents

Suppl.-Table 1. 2

Suppl.-Table 2: 3

Suppl.-Table 3: 4

Suppl. Figure 1: Comparison of antigen specific memory cell phenotypes after stimulation with M, N or S protein SARS-CoV2 OPPs. 6

Suppl. Figure 2. Age comparison between RTx, healthy patients and COVID-19 patients. 7

	CD4 T cells			CD8 T cells		
	Responders	Non-Responders	p-Value	Responders	Non-Responders	p-Value
Age [years][§]	57	53	0.937[#]	53	58	0.908[#]
Sex [female] [φ]	2/13	6/7	**0.004**[$]	4/12	4/8	0.648[$]
Immuno-suppressio						
Tac[§]	4.0	3.0	0.632[#]	3.5	5.25	0.669[#]
Glu [φ]	3/13	3/7	0.617[$]	3/12	3/8	0.619[$]
Cyclosporin A [φ]	0/13	1/7	0.350[$]	0/12	1/8	0.400[$]
MPA [φ]	10/13	4/7	0.613[$]	9/12	4/8	0.642[$]
MMF [φ]	3/13	2/7	1.000[$]	3/12	2/8	1.000[$]
Transplant [living] [φ]	4/13	1/7	0.613[$]	4/12	1/8	0.603[$]
Age of Transplant [month] [§]	70	184	1.000[#]	204	68	0.728[#]
CMV Donor [positive] [φ]	6/13	4/7	1.000[$]	6/12	4/8	1.000[$]
EBV Donor [positive] [φ]	6/11	5/7	0.637[$]	6/11	5/7	1.000[$]
Creatinine [§]	1.27	1.32	0.874[#]	1.27	1.46	0.671[#]
Whole blood count [§]	7.27	7.00	0.526[#]	7.28	7.12	1.000[#]

Suppl.-Table 1. [§] median, [φ] number, [$] Two tailed Fisher's exact Test, [#] Two tailed Man Whitney U Test. Tac=tacrolimus, Glu= Glucocorticoids, MMF=Mycophenolate mofetil, MPA=Mycophenolic Acid.

	RTx	Healthy donors	COVID-19 patients	p-value
Patients [number]	20	20	17	
Age [years]	54.6	56.5	73.5	(Fig. S2)
Sex [female]	8	9	10	ns

Suppl.-Table 2: Differences in sex between all groups were compared by two-tailed Fisher's exact test and differences between ages were compared by Anova and post-hoc T-test (Fig. S3). ns = not significant

RTx Patient	Age	Sex	Immunosuppression	Transpl ant	Age of transplant [days]	Serum Creatinine [mg/dl]	CMV [Donor/ Reciepent]	EBV [Donor/ Reciepent]	WBC [cells/nl]
1	54	m	Tac [2.5], Glu [5], MMF [1000]	living	272	1.21	pos/neg	pos/pos	4.9
2	64	w	Cyclosporin A [100], Glu [5]	living	273	0.82	pos/pos	pos/pos	7
3	43	w	Tac [3], Glu [5], MMF [1000]	cadaver	273	1.09	neg/pos	pos/neg	5.8
4	53	w	Tac [3], Glu [5], MMF [1000]	cadaver	265	1.48	neg/pos	pos/pos	7.4
5	48	m	Tac [1,5], MPA [1440]	cadaver	278	0.99	pos/nd	pos/pos	2.75
6	51	m	Tac [4], MPA [1440]	living	273	1.08	neg/pos	neg/pos	5.60
7	59	m	Tac [2,5], MPA [1440]	cadaver	275	1.26	pos/pos	neg/pos	8.14
8	45	w	Tac [4,5], MPA [720]	cadaver	184	1.64	pos/nd	neg/nd	2.06
9	49	w	Tac [4], MPA [1440]	cadaver	192	1.32	neg/nd	pos/nd	11.8
10	57	w	Tac [17], Glu [15], MMF [2000]	cadaver	60	1.27	neg/nd	na/na	7.3
11	34	m	Tac [7], Glu[15], MMF [2000]	cadaver	53	1.61	neg/nd	na/na	12.1
12	37	m	Tac [3], MPA [1440]	cadaver	216	1.01	neg/nd	pos/pos	6.7
13	71	m	Tac [4], MPA [720]	cadaver	63	1.73	pos/nd	neg/pos	6.91
14	62	m	Tac [6], MPA [1440]	cadaver	62	1.47	pos/nd	pos/pos	7.23
15	44	m	Tac [7], MPA [1440]	living	62	1.55	neg/pos	neg/pos	9.26
16	55	m	Tac [8], MPA [1440]	cadaver	66	2.85	pos/nd	pos/nd	4.02
17	61	m	Tac [6], MPA [1440]	cadaver	63	1.44	neg/nd	neg/nd	11.47
18	61	w	MPA [1440]	cadaver	70	1.19	neg/nd	pos/nd	10.07
19	68	m	Tac [6], MPA [1440]	living	50	1.85	pos/nd	pos/nd	7.27
20	75	w	Tac [3], MPA[1440]	cadaver	58	1.22	pos/nd	neg/nd	7.66

Suppl.-Table 3: Clinical characteristics of patients with renal transplantation. f=female m=male, Tac=tacrolimus (daily dose in mg), Glu= Glucocorticoids (daily dose in mg), MMF=Mycophenolate mofetil (daily dose in mg), MPA=Mycophenolic Acid (daily dose in mg), ne=negative, pos=positive, WBC=white blood count, - = not determined

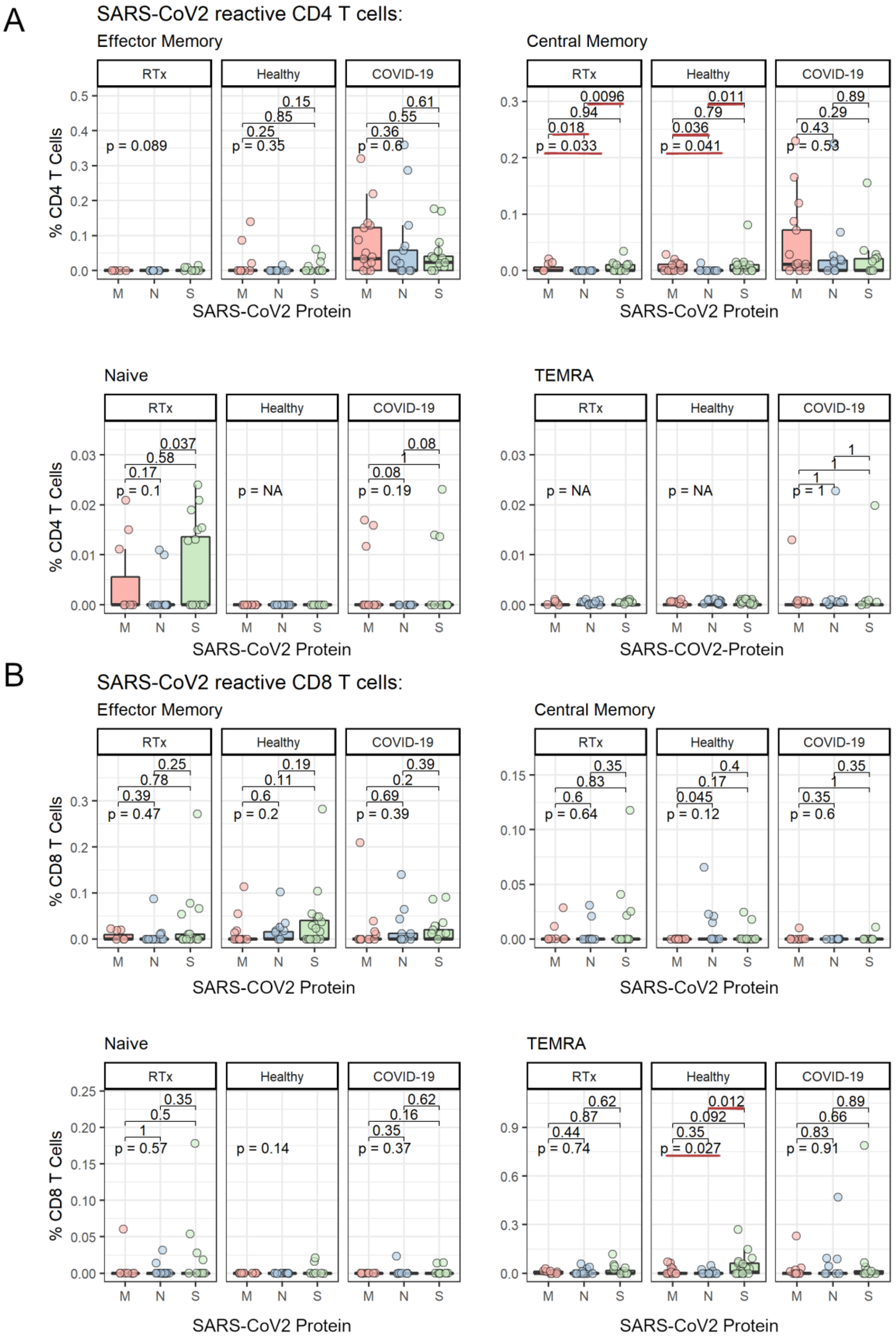

A
SARS-CoV2 reactive CD4 T cells:
Effector Memory
Central Memory
Naive
TEMRA
RTx
Healthy
COVID-19
% CD4 T Cells
SARS-CoV2 Protein
SARS-COV2-Protein
M
N
S
p = 0.089
p = 0.35
p = 0.6
p = 0.033
p = 0.041
p = 0.53
p = 0.1
p = NA
p = 0.19
p = 1
B
SARS-CoV2 reactive CD8 T cells:
Effector Memory
Central Memory
Naive
TEMRA
% CD8 T Cells
SARS-COV2 Protein
SARS-CoV2 Protein
p = 0.47
p = 0.2
p = 0.39
p = 0.64
p = 0.12
p = 0.6
p = 0.57
p = 0.14
p = 0.37
p = 0.74
p = 0.027
p = 0.91

Suppl. Figure 1: Comparison of antigen specific memory cell phenotypes after stimulation with M, N or S protein SARS-CoV2 OPPs. Isolated PBMCs from RTx (20), healthy patients (20) and COVID-19 patients (17) were stimulated for 16h with 1µg/ml SARS-CoV2 OPP from M (n=11-19), N (n=17-20) or S (n=17-20) protein and analyzed by flow cytometry. Comparison of M, N or S reactive memory (A) $CD4^{+}CD137^{+}CD154^{+}$ and (B) $CD8^{+}CD137^{+}$ T cells in RTx patients, healthy donors and COVID-19 patients. Groups were compared using Kruskal-Wallis test (indicated by “p=”); pairwise comparison was done using two-sided, unpaired post-hoc Mann-Whitney-U-Test. p-Values < 0.05 are underlined in red.

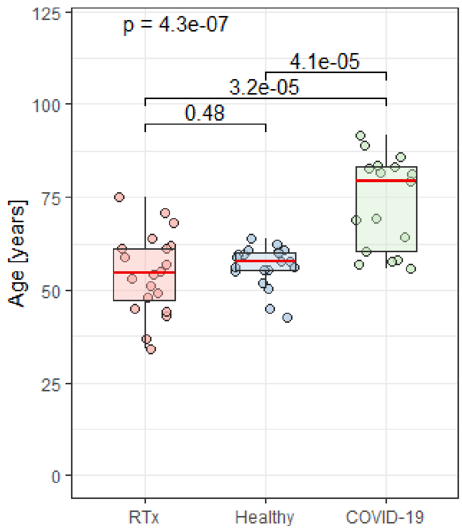

Suppl. Figure 2. Age comparison between RTx, healthy patients and COVID-19 patients. Age of RTx (20), healthy patients (20) and COVID-19 patients (17) compared by Anova (indicated by “p=”) and pairwise comparison using two-sided, unpaired post-hoc T-test.
